# Mental health, gender, and higher education attainment

**DOI:** 10.1007/s11618-023-01187-3

**Published:** 2023-09-06

**Authors:** Kaspar Burger, Diego Strassmann Rocha

**Affiliations:** 1 Jacobs Center for Productive Youth Development & Department of Sociology, University of Zurich, Andreasstrasse 15, 8050 Zurich, Switzerland; 2 Center for Childhood and Youth Research, Department of Social Sciences, University of Luxembourg, Esch-sur-Alzette, Luxembourg; 3 Social Research Institute, Institute of Education, University College London, WC1H 0AL London, United Kingdom; 4 Department of Sociology, University of Zurich, Andreasstrasse 15, 8050 Zurich, Switzerland

**Keywords:** Tertiary education, Risk/resilience, Gender, Sociology, Panel study, Life course, Hochschulbildung, Risiko/Resilienz, Geschlecht, Soziologie, Längsschnittstudie, Lebensverlauf

## Abstract

We compared the mental health of higher education students with that of nonstudents. Moreover, we examined whether the mental health of students predicts their probability of obtaining a higher education degree, and whether the extent to which mental health affects educational attainment varies by gender. Drawing on a risk and resilience framework, we considered five facets of mental health that may be implicated in distinct ways in the educational attainment process: positive attitude towards life, self-esteem, self-efficacy, negative affectivity, and perceived stress. We used data from a nationally representative panel study from Switzerland (*N*_*students*_ = 2070, 42.8% male; *N*_*nonstudents*_ = 3755, 45.9% male). The findings suggest that overall, the mental health of higher education students was relatively similar to that of nonstudents, although students exhibited slightly higher self-esteem, slightly weaker self-efficacy, greater negative affectivity, and higher levels of perceived stress. The effects of different facets of mental health on higher education degree attainment were mostly statistically and/or practically insignificant. However, positive attitudes towards life had a substantial positive effect on the probability of being awarded a higher education degree. Mental health was equally important for male and female students’ educational attainment.

**Supplementary Information:**

The online version of this article (10.1007/s11618-023-01187-3) contains supplementary material, which is available to authorized users.

## Abstract (de)

Wir vergleichen die psychische Gesundheit von Studierenden und Nicht-Studierenden. Zudem untersuchen wir, inwieweit die psychische Gesundheit die Wahrscheinlichkeit eines Hochschulabschlusses bei jungen Männern und Frauen vorhersagt. Auf der Basis eines Risiko- und Resilienz-Modells berücksichtigen wir fünf Dimensionen psychischer Gesundheit, die auf unterschiedliche Weise den Bildungserfolg von Studierenden beeinflussen können – positive Lebenseinstellung, Selbstwert, Selbstwirksamkeit, negative Affektivität und Stresserleben. Wir analysieren Daten aus einer schweizweit repräsentativen Längsschnittstudie (*N*_*Studierende*_ = 2070, 42,8 % männlich; *N*_*Nicht-Studierende*_ = 3755, 45,9 % männlich). Die Befunde deuten darauf hin, dass die psychische Gesundheit von Studierenden und Nicht-Studierenden insgesamt relativ vergleichbar war, wobei Studierende einen leicht höheren Selbstwert, geringfügig geringere Selbstwirksamkeit sowie mehr negative Affektivität und höheres Stressempfinden berichteten. Die meisten Dimensionen psychischer Gesundheit standen in keinem nennenswerten Zusammenhang mit der Wahrscheinlichkeit eines Hochschulabschlusses. Allerdings hatte eine positive Lebenseinstellung einen substantiellen positiven Effekt auf die Wahrscheinlichkeit, einen Hochschulabschluss zu erlangen. Die psychische Gesundheit war für den Bildungserfolg von Männern und Frauen gleichermassen bedeutsam.

## Introduction

Young adults enter a new phase of life that entails new developmental tasks. In early adulthood, life trajectories increasingly diverge, individuals take on new social roles that provide opportunities for them to flourish or fail, and the emergence of new developmental contexts may engender discontinuity in human functioning and adjustment (Schulenberg et al. 2004). Young adults are expected to achieve greater autonomy, assume responsibility for themselves and in the larger community, attain greater emotional maturity, and engage in activities to achieve career goals (Arnett 2014; Bowler and Weinraub 2018; McCormick et al. 2011). Early adulthood thus involves important changes in cognitive and socio-emotional development (Roisman et al. 2004). Against this background, young adults may be particularly prone to developing mental health issues (Kessler et al. 2005).

There has been a particular concern that young adults who pursue higher education might be at even greater risk of mental health issues than their counterparts who do not pursue higher education (Arsandaux et al. 2021; Macalli et al. 2021; Verger et al. 2009). Higher education students have to cope with academic pressure, adjust to new learning environments, and pursue demanding long-term academic goals (e.g., Said et al. 2013). Students are confronted with a particular set of challenges in higher education, in addition to the challenges associated with early adulthood in general. As a result, students in higher education may suffer from high levels of psychological burden (Castillo and Schwartz 2013; Hunt and Eisenberg 2010; Pedrelli et al. 2015; Sarmento 2015), which might ultimately lead to mental health problems (Brown 2018) and impair academic functioning. Studies suggests that approximately one of five students in higher education worldwide has a mental health disorder of some kind (Auerbach et al. 2016). However, there is a paucity of research on whether higher education students exhibit worse mental health than their nonstudent counterparts. There have been a few studies in different national contexts and time periods, and these have provided inconclusive evidence (Arsandaux et al. 2021; McManus and Gunnell 2020; Tabor et al. 2021). Understanding potential differences in students and nonstudents’ mental health is essential—not least to inform public health policy and better allocate resources to improve public mental health (Kovess-Masfety et al. 2016; Tabor et al. 2021).

It is also important to better understand the extent to which mental health is implicated in educational attainment processes. Mental health is associated with human functioning and is crucial for how we think, feel, and act. In educational contexts, good mental health enables students to concentrate, deal with academic challenges, and respond resiliently when confronted with setbacks (Auerbach et al. 2016; Breslau et al. 2011; Evans et al. 2018). Mental health issues may influence students’ capabilities to cope with academic difficulties and may adversely affect their attitudes toward education. Mild mental health conditions may only lead to minimal deficits in social skills in class, minor attention difficulties, vague feelings of perplexity (Fusar-Poli et al. 2018), and fairly inconsequential learning problems (Bernstein et al. 2008). In more severe forms, however, poor mental health may significantly reduce students’ learning capacities and may be seriously disabling; a considerable proportion of students with a psychiatric disorder withdraw from higher education prior to completion of their studies (Kessler et al. 1995).

Mental health seems to be implicated in the dynamics underlying educational attainment processes (Agnafors et al. 2021; Clayborne et al. 2019; Hale and Viner 2018; Mikkonen et al. 2020). However, the influence of mental health on subsequent educational attainment may differ across distinct dimensions of mental health (Breslau et al. 2008; Hale et al. 2015; Mojtabai et al. 2015); for instance, perceived stress might be less consequential than negative affectivity. It is therefore important to disentangle the extent to which different dimensions of mental health may contribute to educational attainment.

Sociological, psychological and gender theories suggest that young men and women tend to have quite different life experiences in some respects; they are also often faced with disparate challenges to their successful acquisition of adult role markers (Burger et al. in press). Young men and women often transition to adulthood by following distinct, societally structured pathways (e.g., Wharton 2009; see also Oesterle et al. 2010; Stoet and Geary 2020). Their pathways to educational attainment vary, and there is now a growing gender gap in tertiary education enrollment and completion, with women far outnumbering men in most economically developed societies (Buchmann et al. 2008). Against this background, the mental health of young men and women might also matter in distinct ways for educational attainment processes.

The role of mental health in educational attainment processes could differ by gender for various reasons. Girls and boys typically experience distinct socialization experiences (e.g., Stockard 2006), which may either exacerbate or attenuate mental health problems. Young men and women usually exhibit different types of mental health impairments (Campbell et al. 2021). They tend to respond differently to specific developmental challenges and distress (Dedovic et al. 2009; Eschenbeck et al. 2007), and there are also gender differences in how individuals cope with mental health issues (Eschenbeck et al. 2007; Wilhsson et al. 2017).

With this in mind, we used data from a nationally representative large-scale panel study from Switzerland to examine (1) the mental health status of higher education students compared to that of nonstudents, (2) the extent to which distinct facets of mental health predict degree attainment among students in higher education, and (3) whether gender moderates the longitudinal links between mental health and degree attainment. It is essential to investigate factors contributing to degree attainment because substantial proportions of students who enter higher education do not subsequently graduate, with estimated dropout rates varying between 10% in Japan, 23% in Germany, and 46% in New Zealand (OECD 2008). Dropout rates were approximately 14% in the Swiss cohort under investigation here (BFS 2012).

### Mental health

Mental health is a unique product of a complex interplay of social and environmental influences, and genetic, neurodevelopmental, and psychological processes. It has been conceptualized as the capacity of thought, emotion, and behavior that enables individuals to realize their own potential relative to their developmental stages, to deal with the typical stresses of life, to study or work productively, and to contribute to their communities (Patel et al. 2018; WHO 2004). Mental health reflects effective psychological functioning and is thought to enable individuals to flourish in life (Keyes 2006), allowing them to adapt to change and deal effectively with environmental demands (Alegría et al. 2015; Manwell et al. 2015).

There is no universal definition of mental health and various indicators have been used to measure mental health (e.g., Breslau et al. 2008; Deighton et al. 2014; Korkeila et al. 2003). Theorists, empirical researchers, and international organizations such as the World Health Organization typically conceive of mental health as a multidimensional concept that includes the presence of multiple human strengths, positive emotions and thoughts, wellbeing, and resilience (Vaillant 2012; WHO 2004)—not merely the absence of mental illness. These different components are resources that constitute the foundation for effective and adaptive human functioning. However, there is no equivalence between such resources and mental health. For instance, positive emotions and subjective wellbeing are not consistently indicative of mental health. There are challenging life situations in which positive emotions and wellbeing may even be regarded as unhealthy. An individual who experiences a state of wellbeing while killing people during a military action would typically be considered as mentally unhealthy; and a person who feels low after being laid off in a labor market with scarce employment opportunities would be considered as mentally healthy (Galderisi et al. 2015). Individuals in good mental health can be unhappy, angry, or distressed, and they may feel overwhelmed by common stressors if they accumulate. Such emotions are part of a fully lived life. Similarly, the idea that mental health must promote effective functioning and community participation has been criticized. Some individuals experience rejection and/or systemic discrimination and are discouraged from contributing to society. Contextual circumstances may prevent some individuals from working productively. Asylum seekers, for instance, may not get permission to work in a given context; as a result, they may lack a sense of mastery and are unable to fully exploit their potential. Their ability to identify, tackle, and overcome problems is impaired as a result of external barriers, not as a result of mental ill-health. Thus, mental health may involve imperfect functioning and emotional states that neither reflect hedonic wellbeing (pleasure attainment and pain avoidance) nor eudaimonic wellbeing (self-actualization and the perception of living a meaningful life; cf. Ryan and Deci 2001).

Scholars agree, however, that mental health implies resilience—the capacity of individuals to adapt to adversity and cope with difficulties in challenging situations or when certain functions have been impaired (see Davydov et al. 2010). With this in mind, mental health can be conceptualized as a resource that enables states of wellbeing and provides individuals with the capacity to achieve their full potential (Patel et al. 2018), though theorists recognize that meaningful functioning and subjective wellbeing can only be achieved when a person has real freedoms in their social environment. Indeed, human functioning and wellbeing can never endure. Everyone occasionally experiences aversive emotional states, has negative thoughts about themselves and the world, and functions less well than normal. Mentally healthy individuals, however, have the ability to bounce back from negative events and thoughts by using positive emotions and thoughts to cope. They exhibit functional adaptation when faced with challenges and risks; they manage to live their lives even when these lives are complicated, using their resources and capabilities to reduce the odds of undesirable outcomes (Panter-Brick 2014).

Risk and resilience models of mental health reflect this principle—mentally healthy individuals experience risks, but they are better protected against harm and are able to modulate their emotions and function at higher levels when faced with adversity (Galderisi et al. 2015; see also Tugade et al. 2004). Environmental demands can jeopardize an individual’s response capability, but resilient individuals use regulatory processes and socio-cognitive resources to cope with challenges (Masten 2004; see also Lazarus and Folkman 1984). Consistent with such a risk and resilience model, we considered five psychological and (socio)cognitive dimensions which we consider as either resilience or risk factors for mental health: an individual’s attitude towards life, self-esteem, and self-efficacy (resilience factors), as well as negative affectivity and perceived stress (risk factors). These dimensions of mental health are assumed to enable or undermine adaptive psychological and behavioral functioning; as such, they may be fundamental to educational attainment processes (Pascoe et al. 2020; Patalay and Fitzsimons 2018; Smith et al. 2021).^1^

### Facets of mental health: how they may shape educational attainment

Different psychological and (socio)cognitive dimensions may be implicated in distinct ways in educational attainment processes.

#### Positive attitude towards life

An individual’s attitude towards life refers to their overall evaluation of their life. It is a by-product of cognition, affect, and behavior (Kato et al. 2016), and reflects the belief that life is working out well. Thus, it is an important cognitive component of optimism, which typically prompts individuals to direct their efforts into goal-oriented and sustained action (Burger and Mortimer 2021). Empirical evidence indicates that a positive attitude towards life may influence various behavioral outcomes (Albarracin and Shavitt 2018; Glasman and Albarracín 2006; Maio et al. 2018). Consequently, it might also influence educational attainment.

#### Self-esteem

Self-esteem refers to how individuals subjectively evaluate and view themselves (Orth et al. 2012)—how they judge their self-worth. Self-esteem is typically conceptualized as an element of self-concept (Cast and Burke 2002). Individuals’ evaluations of themselves can entail positive and negative components, ranging from self-confidence to self-deprecation (Rosenberg et al. 1995). Self-esteem is not necessarily an objective reflection of an individual’s abilities and talents, or even of how an individual is viewed by others. Instead, it involves feelings of self-regard and self-acceptance (Orth and Robins 2014). Individuals with high self-esteem commonly feel good about themselves. They feel that they have various good qualities (Robins and Trzesniewski 2005) and have a positive attitude towards themselves.

There is an ongoing debate on whether people with high self-esteem have better life prospects than those with low self-esteem. A positive self-image may influence how individuals act in a broad range of contexts, potentially affecting motivation and goal-setting as well as success and failure in various domains. For instance, Trzesniewski et al. (2006) observed that adolescents who exhibited low self-esteem had worse economic prospects, poorer health, higher levels of school dropout, and more criminal behavior during young adulthood than adolescents with high self-esteem. Similarly, studies have found that self-esteem is related to greater job performance and more successful work careers (Judge and Bono 2001; Judge and Hurst 2008). Self-esteem may also lead to better educational performance and higher educational attainment (Booth and Gerard 2011; Marsh and O’Mara 2008). Typically, however, researchers have only found weak longitudinal associations between self-esteem and later outcomes (Boden et al. 2008), and some scholars have criticized claims that self-esteem has any substantial influence on individuals’ later life courses (Baumeister et al. 2003). One reason for the inconclusive nature of the evidence is that studies on the predictive power of self-esteem have focused on quite disparate outcomes, ranging from affect and depression to trajectories of relationships and occupational status (Leary and Baumeister 2000; Orth et al. 2012; Swann et al. 2007). It is therefore essential to specifically analyze whether self-esteem plays a role in shaping higher education degree attainment.

#### Self-efficacy

Self-efficacy refers to how individuals evaluate their capacity to engage in specific behaviors, achieve a given aim, or exercise control over an action or specific events (Bandura 2006). Self-efficacy is a key dimension of self-concept, and it may be an essential resource facilitating attainment processes (Abele and Spurk 2009). It is typically related to goal setting, perseverance, and the capacity to deal with setbacks and failures in a constructive manner (Samuel and Burger 2020; Schwarzer 2000). Individuals who feel more efficacious report greater levels of confidence in their capabilities and lower levels of test anxiety in academic settings (Nie et al. 2011). They are also more likely to perform better academically (Burger et al. 2020; Burger and Walk 2016). Therefore, self-efficacy may also matter for educational attainment processes.

#### Negative affectivity

Negative affectivity refers to a stable tendency to experience aversive emotional states (Watson and Clark 1984). Individuals who exhibit high levels of negative affectivity tend to report unpleasant mood states at all times, regardless of the situation (Denollet 2013). They have an attention bias toward adverse stimuli, taking a gloomy view of things and showing an inclination to feel concerned and irritable. Their depressed moods are frequently accompanied by other aversive affective states like anger, anxiety, and guilt (Watson and Pennebaker 1989). Negative affectivity has been regarded as a risk factor for various health problems, as it is associated with a range of issues such as depression and anxiety disorders (Norton and Paulus 2016), neuroticism (Watson and Naragon-Gainey 2014), lower levels of social functioning (Sanmartín et al. 2018), and school-related stress (Hirvonen et al. 2019). Negative affectivity might therefore also lead to poor educational performance. So far, however, only one study has investigated associations between negative affectivity and school performance. This study found no evidence of significant associations between the two (Sanmartín et al. 2018). However, negative affectivity and school performance were measured simultaneously; whether negative affectivity has longer-term implications for attainment remains to be investigated.

#### Perceived stress

Perceived stress refers to a negative psychological reaction to a challenging experience (or stressor). Such reactions often ensue when demands exceed a person’s resources or capabilities to cope with and respond to a stressor (Lazarus 2006). Perceived stress can include a range of cognitive and affective states, such as the sense of being overwhelmed, sorrow, and anxiety (Folkman 2013; Hampel and Petermann 2006). Various aspects of stressors can influence the severity of the stress reaction, including the stressor’s duration or controllability and the extent of the demands (Kemeny 2003). Importantly, while stressful experiences may be perceived as straining, positive emotions may co-occur with negative emotions during stressful situations and may have a restorative function in psychological and physiological terms (Folkman 2008). By extension, stressful experiences may lead to positive personal change and stress-related personal growth, with the result that individuals can ultimately benefit from the stressful experience (Bower et al. 2008; Park et al. 1996).

In educational contexts, students are confronted with a wide array of demands that they may experience as taxing. Perceived stress in education may influence students’ sleep quality, physical health, and ability to concentrate (Pascoe et al. 2020), limiting their learning capacity and potentially impairing educational achievement (Gustems-Carnicer et al. 2019; Rothon et al. 2009; Vaez and Laflamme 2008). Moreover, exceptionally stressful life events are risk factors for school dropout (Dupéré et al. 2018; Samuel and Burger 2020). Hence, perceived stress might also affect students’ likelihood of attaining a higher education degree.

### Mental health and educational attainment: considering theories of gender

Gender theorists emphasize that the lives of young men and women may differ substantially in various domains. Disparities in the life experiences of young men and women are mostly the result of unequal socialization experiences, social norms, cultural values and ideologies, institutional arrangements, political trends, economic and welfare systems, and other macro-structural forces (e.g., Chafetz 2006). In many societies, there are prototypical gender divisions of labor, unequal pathways to adulthood, gendered forms of civic participation, and diverging normative expectations regarding male and female roles in society (Orloff 1996). Gender-typical patterns of thoughts, feelings, and behaviors are mainly shaped culturally and develop over time (Jule 2014; see also Martin 2004). In the long term, they may lead to considerable gender inequalities in mental health, behavior, and attainment (e.g., Bambra et al. 2009; Palència et al. 2014).

With this in mind, we consider whether any potential links between mental health and educational attainment might vary by gender. Gender might moderate the influence of mental health on educational attainment because of gender-typical patterns of thoughts and behaviors (Jule 2014), because young men and women tend to suffer from different types of mental health issues (Riecher-Rössler 2017), because they respond in different ways to developmental challenges (Dedovic et al. 2009; Eschenbeck et al. 2007), or because they exhibit gender-specific strategies to cope with mental health issues (Eschenbeck et al. 2007; Wilhsson et al. 2017).

Studies have shown that young women tend to experience higher levels of stress than men and their coping style is sometimes more emotion-centered (Matud 2004). Similarly, some studies have indicated that young women may be particularly vulnerable because they react less adaptively to stressors than young men (Rosenfield and Mouzon 2013). This suggests that poor mental health might particularly harm educational attainment processes among young women. A number of studies have provided evidence consistent with this assumption, showing that specific mental health problems are especially related to lower educational attainment among female students. Internalizing problems during adolescence tend to be associated with lower educational attainment in young adulthood, but only among young women (Veldman et al. 2014). Moreover, studies have indicated that depressive symptomatology during adolescence is only associated with an increased likelihood of failing to obtain a high school diploma (Needham 2009) and not enrolling in college (Fletcher 2008) among female students. Finally, studies have found that links between impaired mental health and poor educational outcomes in young adulthood are stronger among young women than men (Cornaglia et al. 2015).

Overall, however, the empirical evidence is inconclusive; other studies have suggested that poor mental health tends to disadvantage both female and male students in terms of their educational attainment. Veldman et al. (2015) found that externalizing problems predicted lower educational attainment in young adulthood among both genders. Von Simson et al. (2022) reported that the same was true for internalizing problems. Using more global measures of mental health, Hjorth et al. (2016) identified links between poor mental health and increased odds of subsequently dropping out of higher education among both young men and women. Finally, Smith et al. (2021) also found evidence suggesting that mental health difficulties predicted lower levels of educational attainment exclusively among male students.

Taken together, the empirical findings regarding gender differences in the links between mental health and subsequent educational outcomes are inconsistent (Brännlund et al. 2017; Deighton et al. 2018; Ding et al. 2009; Esch et al. 2014; Wickersham et al. 2021). This may be explained in part by the different ways that different studies have operationalized mental health, with some studies using a single score to assess mental health rather than adopting a multidimensional perspective. With this in mind, we examined whether distinct facets of mental health predicted higher-education degree attainment, and whether gender moderated any associations between those facets of mental health and later degree attainment.

### The present study

This study pursued three main goals. *First*, we assessed whether the mental health of higher education students differed from that of their nonstudent counterparts, taking into account potential gender differences. This allowed us to gauge the extent to which students in higher education might be dealing with mental health problems specific to them. *Second*, we examined the degree to which mental health predicted higher-education students’ probability of obtaining a degree at this level. We conceptualized mental health as a multidimensional construct within a risk and resilience framework, examining individuals’ attitudes towards life, self-esteem, self-efficacy, perceived stress, and negative affectivity. *Third*, we considered whether the role of these dimensions of mental health for higher education degree attainment varied by gender. Specifically, we addressed three research questions.

#### Research question 1

Does the mental health status of (male and female) higher education students differ from that of nonstudents?

#### Research question 2

To what extent do distinct dimensions of mental health predict degree attainment among higher education students?

#### Research question 3

Does gender moderate the longitudinal links between distinct dimensions of mental health and higher education degree attainment

We expected that the mental health of students might differ from that of nonstudents; students are confronted with a particular set of challenges in higher education which might lead to psychological burden and mental health impairments. Furthermore, we expected that distinct dimensions of mental health would significantly predict the likelihood of attaining a higher education degree. However, we refrained from formulating specific hypotheses regarding the effects of particular dimensions of mental health on degree attainment, as prior research into this question has provided inconsistent findings. Finally, we presumed that gender might moderate the links between distinct dimensions of mental health and higher education degree attainment, although prior studies on this topic have yielded divergent results, preventing us from formulating a more specific (directional) hypothesis. To address our research questions, we used data from a large-scale panel study that followed a cohort of individuals born around 1985 in Switzerland and tracked their educational and labor market trajectories until around age 30.

## Method

### Data

We drew on data from a nationwide study—the Transitions from Education to Employment (TREE) panel survey—that has followed a cohort of individuals since 2001. All research procedures performed during data collection conformed with ethical standards and applicable laws. All data are publicly available at SWISSUbase (10.23662/FORS-DS-816-7) and are entirely de-identified. An examination by the Institutional Review Board of the University of Zurich was not required for the present secondary data analysis. Materials and analysis code for this study are available by emailing the corresponding author. In the present study, we follow state-of-the-art article reporting standards (Kazak 2018) and explain all data selection procedures and data manipulations and describe all measures used hereafter.

The TREE sample consisted of 6343 respondents who initially participated in the Program for International Student Assessment (PISA) during their last year of lower-secondary school (in the year 2000).^2^ The TREE panel was conducted between 2001 and 2014. From 2001 to 2007, panel waves were carried out each year (t_1_ to t_7_). Two additional waves were conducted in 2010 and 2014 (t_8_ and t_9_). To assess the mental health status of higher education students and nonstudents, we considered all respondents for whom information was available on whether or not they participated in higher education at any given point during the observation period (2001–2014). This analytic sample included 5825 respondents. Within this analytical sample, 2070 individuals were enrolled in higher education at some point, whereas 3755 individuals were never enrolled in higher education.

The entire analytic sample (*N* = 5825) differed only very marginally from the original sample (*N* = 6343). Relative to the original sample, the analytic sample included slightly smaller proportions of men (44.8% versus 45.8%) and immigrants (13.6% versus 14.3%). Yet, it was almost identical to the original sample in terms of respondents’ age (*M* = 15.5 years, *SD* = 0.7 versus *M* = 15.5 years, *SD* = 0.7) and parental socioeconomic status (*M* = 50.5, *SD* = 16.2 versus *M* = 50.4, *SD* = 16.3). Finally, the two samples differed in the level of educational performance by less than 0.04 standard deviations, as indicated by the PISA reading test score measured at age 15 (*M*_analytic_ = 513.6, *SD* = 87.4 versus *M*_original_ = 510.01, *SD* = 89.0).

### Measures

Data were collected via questionnaires that were sent to study participants (in panel waves 1 to 4), and with both computer-assisted telephone interviews and questionnaires (in waves 5 to 9). Most of the data collection took place between April and June in each wave. The measures used in this study are described hereafter. Note that we assessed respondents’ mental health over a three-year period, from 2005 to 2007, which corresponds to the period during which the vast majority of respondents who ever enrolled in higher education pursued their initial three years of higher education. We first calculated mental-health scale means for each wave (2005–2007) and then averaged the scale means across the three waves. Table 1 shows the descriptive statistics separately for the individuals pursuing higher education and for those *not* pursuing higher education. Table 2 shows descriptive statistics for each group separately by gender. Table 3 reports the correlations among all variables for the sample of individuals who pursued higher education.

**Table 1:** Descriptive Statistics

		Individuals in higher education					Individuals *not* in higher education				
	Collected in	N	Mean	SD	Min	Max	N	Mean	SD	Min	Max
Male	2000	2070	0.43	0.50	0.00	1.00	3755	0.46	0.50	0.00	1.00
Immigrant	2000	2062	0.09	0.29	0.00	1.00	3729	0.16	0.37	0.00	1.00
Age (in months)	2000	2065	184.35	7.20	161.00	219.00	3744	187.32	7.93	142.00	228.00
Socioeconomic status (HISEI)	2000	1929	57.50	15.93	16.00	90.00	3442	46.64	15.03	16.00	90.00
Reading performance at age 15	2000	2069	564.58	69.24	257.70	884.49	3751	485.47	83.59	27.60	812.88
Math performance at age 15	2000	1179	584.06	78.20	202.14	815.90	2046	514.34	87.62	202.14	811.84
Science performance at age 15	2000	1132	562.32	81.55	168.60	830.09	2102	482.13	90.05	168.60	804.54
Academic self-concept at age 15	2000	2034	0.25	0.91	−2.51	1.84	3639	−0.15	0.85	−2.51	1.84
Study effort at age 15	2000	2035	0.18	0.97	−3.12	2.20	3677	−0.08	0.97	−3.12	2.20
Control expectation at age 15	2000	2035	0.27	0.91	−3.38	2.24	3679	−0.05	0.92	−3.38	2.24
Higher education enrollment	2001–2014	2070	1.00	0.00	1.00	1.00	–	–	–	–	–
University^a^	2001–2014	2070	0.56	0.50	0.00	1.00	–	–	–	–	–
Higher education attainment	2001–2014	1762	0.83	0.38	0.00	1.00	–	–	–	–	–
Positive attitude towards life	2005–2007	1931	4.83	0.71	1.40	6.00	2161	4.84	0.74	1.00	6.00
Self-esteem	2005–2007	1930	4.21	0.54	1.75	5.00	2161	4.16	0.53	1.00	5.00
Self-efficacy	2005–2007	1931	3.10	0.41	1.50	4.00	2161	3.12	0.41	1.00	4.00
Negative affectivity	2005–2007	1931	2.28	0.69	1.00	4.60	2156	2.22	0.70	1.00	5.00
Perceived stress	2005–2007	1789	2.71	0.73	1.00	5.00	724	2.21	0.76	1.00	4.80

We used data from the entire observation period (2001–2014) to assess whether respondents were ever enrolled in higher education. Most respondents attended higher education starting in 2005. Thus, we assessed mental health over a three-year period, from 2005 to 2007, in the years when most respondents who ever attended higher education did so. Thirty-nine respondents were excluded because they reported having obtained a degree from higher education although they were never enrolled in higher education. Our measure of perceived stress captured education-related stress; hence, among individuals who did not pursue higher education, perceived stress referred to other types of education (i.e., non-tertiary education). Consequently, only a small proportion of those individuals reported their perceived stress levels. ^a^Reference category = university of applied sciences or university of teacher education. For respondents who changed the type of higher education (switching, for example, from a university of applied sciences to a conventional university), the latest response was considered

**Table 2:** Means and Standard Deviations of Individual Mental Health Dimensions (Rescaled to a Range from 0 to 5)

	Individuals in higher education			Individuals *not* in higher education		
	All	Female	Male	All	Female	Male
	M (SD)	M (SD)	M (SD)	M (SD)	M (SD)	M (SD)
Positive attitude towards life	3.83 (0.71)	3.81 (0.70)	3.86 (0.72)	3.84 (0.75)	3.81 (0.75)	3.87 (0.76)
Self-esteem	4.00 (0.68)	3.95 (0.70)	4.08 (0.64)	3.95 (0.67)	3.90 (0.68)	4.02 (0.65)
Self-efficacy	3.50 (0.68)	3.38 (0.68)	3.64 (0.66)	3.54 (0.68)	3.44 (0.67)	3.65 (0.68)
Negative affectivity	1.61 (0.86)	1.73 (0.87)	1.44 (0.81)	1.53 (0.88)	1.63 (0.90)	1.43 (0.84)
Perceived stress	2.14 (0.92)	2.18 (0.93)	2.10 (0.90)	1.53 (0.95)	1.56 (0.94)	1.48 (0.95)

*M* Mean, *SD* Standard deviation

**Table 3:** Correlation Matrix

	1	2	3	4	5	6	7	8	9	10	11	12	13	14	15	16
1 Male	–	–	–	–	–	–	–	–	–	–	–	–	–	–	–	–
2 Immigrant	−0.01	–	–	–	–	–	–	–	–	–	–	–	–	–	–	–
3 Age (in months)	0.04	0.06*	–	–	–	–	–	–	–	–	–	–	–	–	–	–
4 Socioeconomic status (HISEI)	0.03	0.00	−0.06**	–	–	–	–	–	–	–	–	–	–	–	–	–
5 Reading performance at age 15	−0.13***	−0.12***	0.08***	0.11***	–	–	–	–	–	–	–	–	–	–	–	–
6 Math performance at age 15	0.20***	−0.14***	0.10***	0.09**	0.46***	–	–	–	–	–	–	–	–	–	–	–
7 Science performance at age 15	0.13***	−0.11***	0.03	0.14***	0.56***	0.42***	–	–	–	–	–	–	–	–	–	–
8 Academic self-concept at age 15	0.04*	0.00	−0.06**	0.07**	0.22***	0.21***	0.17***	–	–	–	–	–	–	–	–	–
9 Study effort at age 15	0.00	−0.02	0.11***	−0.01	0.05*	0.02	0.04	0.31***	–	–	–	–	–	–	–	–
10 Control expectation at age 15	0.10***	0.02	−0.08***	0.06**	0.05*	0.13***	0.11***	0.52***	0.43***	–	–	–	–	–	–	–
11 University	0.00	0.04	−0.12***	0.15***	0.13***	0.09**	0.15***	0.15***	0.07**	0.14***	–	–	–	–	–	–
12 Higher education attainment	−0.06*	−0.01	−0.04	0.07**	0.09***	0.06	0.07*	0.08***	0.01	0.04	0.02	–	–	–	–	–
13 Positive attitude towards life	0.02	−0.04	0.06*	0.00	0.03	0.01	0.00	0.12***	0.11***	0.09***	−0.05*	0.10***	–	–	–	–
14 Self-esteem	0.09***	−0.05*	0.01	0.05*	0.10***	0.11***	0.15***	0.18***	0.07**	0.17***	0.02	0.09***	0.65***	–	–	–
15 Self-efficacy	0.20***	−0.01	0.07**	0.01	0.11***	0.12***	0.20***	0.17***	0.09***	0.21***	0.02	0.02	0.52***	0.57***	–	–
16 Negative affectivity	−0.18***	0.11***	−0.19***	−0.02	−0.15***	−0.24***	−0.18***	−0.12***	−0.07**	−0.03	0.03	−0.06*	−0.46***	−0.39***	−0.34***	–
17 Perceived stress	−0.05*	0.04	−0.07**	0.00	−0.08***	−0.10**	−0.08**	0.00	0.00	0.00	0.14***	0.00	−0.22***	−0.21***	−0.22***	0.28***

Correlations based on the sample of individuals pursuing higher education (*N* = 2070). Pearson coefficients are reported for correlations between continuous variables, point-biserial coefficients for correlations that include a dichotomous variable, and Phi coefficients for correlations between two binary variables. To test for potential multicollinearity, we estimated variance inflation factors (VIF). The VIF values ranged between 1.03 (for socioeconomic status (HISEI)) and 2.10 (for positive attitude towards life)

***p < 0.001, **p < 0.01, *p < 0.05

#### Positive attitude towards life

Attitude towards life was measured on a scale that consisted of six items, adapted from a questionnaire on youth wellbeing (Grob et al. 1991). The Likert-type rating scale ranged from 1 (totally wrong) to 6 (exactly right). The items were: “My future looks good,” “I am satisfied with how my life plans are getting fulfilled,” “Whatever happens, I can see its positive side,” “I like to live,” “I can cope well with things that I cannot change in my life,” “My life seems meaningful to me.” Cronbach’s alpha varied between 0.85 and 0.87 across the three waves considered here (*t*_2005_: 0.85, *t*_2006_: 0.87, *t*_2007_: 0.87).

#### Self-esteem

Self-esteem was measured with a scale that consisted of five items, adapted from Rosenberg (1979). The Likert-type rating scale ranged from 1 (not at all true) to 4 (very true). Respondents were asked to answer the question “How do you see yourself?” This was followed by the items “On the whole, I am satisfied with myself,” “I feel that I have a number of good qualities,” “I am able to do things as well as most other people,” “I feel that I am a person of worth, at least on par with others,” “I have a positive attitude towards myself.” Cronbach’s alpha varied between 0.80 and 0.81 (*t*_2005_: 0.81, *t*_2006_: 0.81, *t*_2007_: 0.80).

#### Self-efficacy

Perceived self-efficacy was measured with a scale that consisted of four items, adapted from Schwarzer and Jerusalem (1999) and Schwarzer (2000). The Likert-type rating scale ranged from 1 (completely wrong) to 4 (completely right). The items were: “I can find a solution to any problem,” “Whatever happens, I will handle any difficult situation,” “When a problem arises, I can always find a solution by my own efforts,” and “I am confident that I can cope with difficult challenges because I can trust my abilities.” Cronbach’s alpha varied between 0.75 and 0.76 (*t*_2005_: 0.76, *t*_2006_: 0.75, *t*_2007_: 0.76).

#### Negative affectivity

Negative affectivity was measured with a scale that consisted of five items, adapted from Krohne et al. (1996). The Likert-type rating scale ranged from 1 (not at all) to 4 (very much). Respondents were asked to answer the question: “Over the last month, how did you generally feel?” This was followed by the items “irritable,” “concerned,” “annoyed,” “anxious,” and “guilty.” Cronbach’s alpha varied between 0.78 and 0.79 (*t*_2005_: 0.78, *t*_2006_: 0.79, *t*_2007_: 0.78).

#### Perceived stress

Perceived stress was measured with a scale that consisted of five items, adapted from job analysis instruments (Dunckel 1999; Prümper et al. 1995). The Likert-type rating scale ranged from 1 (very seldom/never) to 5 (very frequently/always). The scale assessed education-related stress using the following items: “At school I often feel out of my depth,” “I have too much to do at school,” “The subjects of the lessons change so quickly that I struggle to follow,” “I am often unable to cope with my homework,” and “If I do not study during the weekends, I can hardly manage what is asked of me at school.” It is important to note that these items captured education-related stress. Hence, among individuals who did not pursue higher education, perceived stress referred to other types of education (i.e., non-tertiary education). Consequently, only 19.3% of those individuals reported their perceived stress levels. Cronbach’s alpha for the entire sample varied between 0.83 and 0.85 (*t*_2005_: 0.85, *t*_2006_: 0.85, *t*_2007_: 0.83).

#### Gender

We used a binary variable to measure gender (0 = female, 1 = male).

#### Higher education degree

We used a binary variable to measure whether a study participant had ever obtained a higher education degree in the entire observation period from 2001 to 2014, that is, the period during which virtually all students who ever completed higher education, did so. Specifically, we considered degrees from universities of applied sciences and universities of teacher education as well as bachelor and master’s degrees (and licentiate certificates, the equivalents of master’s degrees) from universities.

#### Covariates

To control for observable potential confounders, we used the following covariates.

##### Socio-demographics

We controlled for age (in months) and immigrant background (0 = born in Switzerland, 1 = born abroad).

##### Highest parental socioeconomic status (HISEI)

Parental socioeconomic status was measured using the standard International Socio-Economic Index of Occupational Status (ISEI) scale (Ganzeboom et al. 1992). This occupation-based measure of socioeconomic status has been used extensively in research because in industrialized societies, a person’s occupation reflects their place in that society and accordingly their social standing and income (Galobardes et al. 2006). Given that each occupational activity requires a given level of education and is remunerated correspondingly, the ISEI score indicates not only a person’s standing in an occupational hierarchy but also proxies their educational background and income (Connelly et al. 2016). The ISEI scale ranges from 16 to 90 and includes a wide variety of occupations (ranging from agricultural workers such as farmers to lawyers and scientists). Where the scores of fathers and mothers differed, we considered the higher score, which is typically referred to as the HISEI score.

##### Student performance

We controlled for the PISA test scores in reading, math, and science (Adams and Wu 2002). These scores were assessed during study participants’ last year of lower-secondary education (in ninth grade, in the year 2000). They reflect respondents’ achievement levels at around age 15.

##### Academic self-concept

We controlled for respondents’ academic self-concept using an index variable from PISA that consisted of three items: “I learn things quickly in most school subjects,” “I’m good at most school subjects,” and “I do well in tests in most school subjects.”

##### Study effort

We controlled for respondents’ study effort using an index variable from PISA that consisted of four items: “I work as hard as possible,” “I keep working even if the material is difficult,” “I try to do my best to acquire the knowledge and skills taught,” and “I put forth my best effort.”

##### Control expectation

We controlled for respondents’ control expectation using an index variable from PISA that consisted of four items: “When I sit myself down to learn something really difficult, I can learn it,” “If I decide not to get any bad grades, I can really do it,” “If I decide not to get any problems wrong, I can really do it,” and “If I want to learn something well, I can.”

##### University education

We used a binary variable to assess whether a study participant attended a traditional university (coded 1) or else a university of applied sciences or university of teacher education (coded 0). We distinguished between the two types of universities because dropout rates and, consequently, degree attainment rates might differ across institutions.

### Data analyses

#### Missing data

Most panel studies have some missing data, which may limit the generalizability of findings. In our dataset, the percentage of missing data due to item nonresponse ranged from 0.0 to 7.9% for the sociodemographic and educational performance measures (1.8% on average), from 21.5 to 33.4% for the higher education enrollment, type of higher education, and higher education attainment variables (25.5% on average), and from 19.7 to 62.6% for the mental health variables (28.4% on average across items and waves). Missingness was predictable through a range of variables that we included in the analytic models. Thus we ran our models based on the missing at random (MAR) assumption. When missing values are related to observed variables included in the models, as was the case here, principled missing data treatments are to be preferred over traditional approaches such as listwise or pairwise deletion or mean substitution (Little et al. 2014). Thus, to minimize estimation bias resulting from missing data, we employed multiple imputation, replacing missing values with plausible imputed values that were generated in an iterative approach using regression techniques. Each imputed value includes a random component whose size represents the degree to which other variables in the imputation model fail to predict its true value. Multiple imputation retains the variance in the data and associations among variables and enables a more precise estimation of parameters. This makes it an ideal and widely-used procedure for dealing with missing data (Baraldi and Enders 2020; Graham 2009). For each missing value, we estimated 25 imputed values, each based on five iterations, using multivariate imputation by chained equations (MICE). We thereby accounted for the uncertainty that is associated with missing data, and reproduced the variance/covariance matrix that we would have observed if the data source had not had any missing data (Lang and Little 2018; Young and Johnson 2015). The MICE procedure uses predictive mean matching and logistic regression techniques to impute continuous and binary data, respectively. We followed the common recommendation of using the complete set of study variables in the imputation model (Graham 2009). We used Rubin’s (1987) rule to pool the coefficient estimates and standard errors resulting from our analyses across datasets.

#### Analytic approach

We estimated multivariate analyses of variance, including post hoc analyses with *p-*values adjusted for multiple testing using the method proposed by Benjamini and Hochberg (1995), to assess whether the mental health of higher education students differed from that of their nonstudent counterparts. We also considered gender differences in the mental health of students and nonstudents to improve our understanding of the mental health among both groups. Moreover, we estimated linear probability models to assess students’ probabilities of attaining a higher education degree as a function of mental health and gender. We controlled for the type of higher education that students pursued (university or university of applied sciences/teacher education), sociodemographic characteristics (age, highest parental socioeconomic status, and immigrant background), student performance at age 15 (using the PISA reading, math and sciences scores), and self-appraisals as well as motivational disposition at age 15 (academic self-concept, control expectation, and study effort). Our models generated coefficients that reflect the conditional average change in the probability of degree attainment that is associated with a one-unit increase in a given predictor. Linear probability models are advantageous because they yield parameter estimates that can be interpreted directly in terms of probabilities across different groups (Angrist and Pischke 2009). We produced standard errors that are robust to the nonindependence of observations and nonnormal distributions, as required for linear probability models (Gomila 2021). We report linear probability coefficients and, moreover, present predicted probabilities, depicting those probabilities using graphs to provide an additional measure of effect size. As a sensitivity analysis, we also estimated all models using a nonlinear (logistic) specification. Appendix A reports the results from these models.

We performed all statistical analyses and created all figures in the *R* statistical environment, version 4.0.2 (R Core Team 2020), using base *R* functions as well as the packages *dplyr*, version 1.0.7 (Wickham et al. 2021), *mice*, version 3.13.0 (van Buuren et al. 2021), *sjPlot*, version 2.8.9 (Lüdecke et al. 2021), *stargazer*, version 5.2.1 (Hlavac 2018), and textreg, version 0.1.5 (Miratrix 2018).

It is widely recognized that statistical power depends on multiple aspects, including the model that is estimated, the distributions of the variables used in the model, and the proportions of missing data present in the data source (Wolf et al. 2013). Yet multiple prior studies that estimated various analytic models based on the TREE sample or subsamples detected significant regression coefficients with tendencies toward small standard errors (e.g., Burger 2021, 2023; Combet and Oesch 2020; Keller et al. 2015). This indicates that the current sample is sufficiently large for a wide range of analyses such as the models estimated here.

## Results

### Mental health of higher education students and nonstudents

With respect to *Research Question 1*, we first estimated a multivariate analysis of variance, using variables that were rescaled to range from 0 to 5 (Table 2 reports the respective descriptive statistics). This analysis revealed that the mental health of higher-education students differed significantly from that of their nonstudent counterparts, *F* (5, 6298) = 140.61, *p* < 0.001. Post hoc analyses using *p*-values adjusted for multiple testing indicated that the average level of self-esteem was slightly but significantly higher among individuals who pursued higher education than among those who did not (*M* = 4.00, *SD* = 0.68 versus *M* = 3.95, *SD* = 0.67, *p* < 0.05). Moreover, self-efficacy was slightly but significantly lower among students than among nonstudents (*M* = 3.50, *SD* = 0.68 versus *M* = 3.54, *SD* = 0.68, *p* < 0.05). The level of negative affectivity was significantly higher among students than among nonstudents (*M* = 1.61, SD = 0.86 versus *M* = 1.53, *SD* = 0.88, *p* < 0.001). Perceived stress was significantly higher among students than among nonstudents (*M* = 2.14, *SD* = 0.92 versus *M* = 1.53, *SD* = 0.95, *p* < 0.001). Finally, students and nonstudents did not differ significantly in terms of their attitudes towards life (*p* > 0.05). In sum, the results suggested that the mental health status of students was substantially quite similar to that of nonstudents. The most notable difference was found in the level of perceived stress, which was considerably higher among students than nonstudents, but it should be noted that our measure of perceived stress captured education-related stress; hence, among individuals who did not pursue higher education, the measure captured perceived stress in some other (non-tertiary) education and, as such, applies only to a subgroup of individuals.

**Fig. 1:**
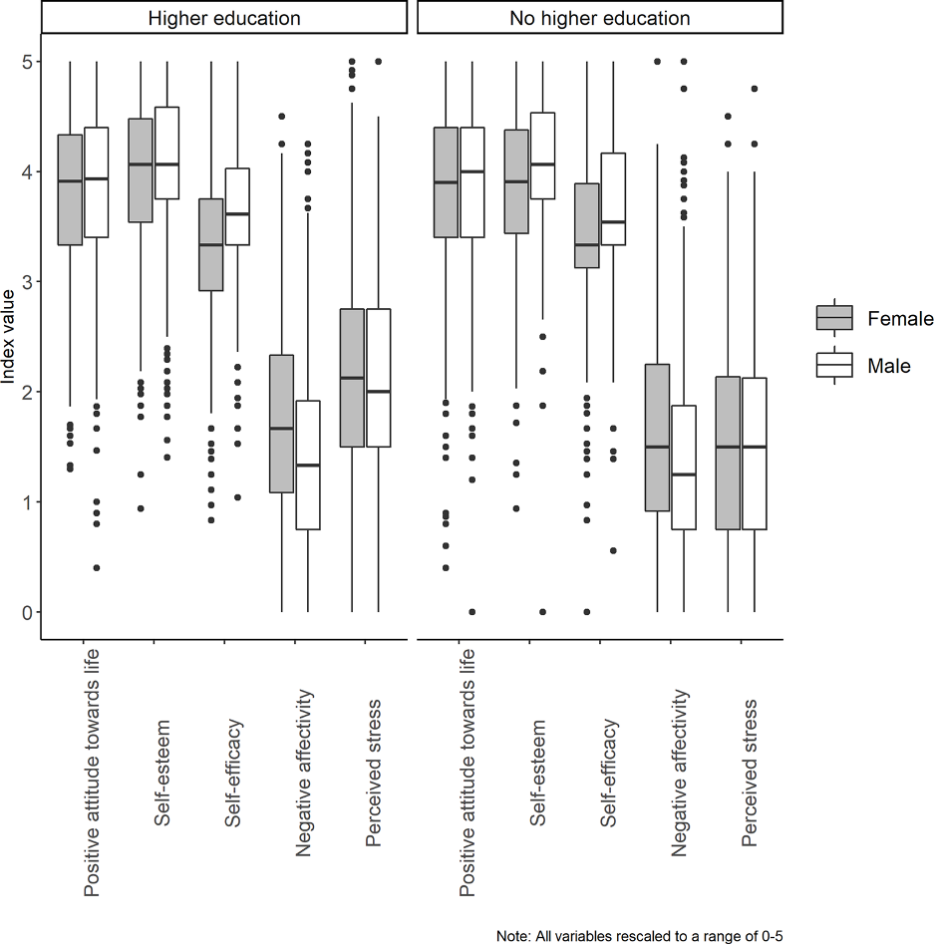
Box plots of the distribution of distinct dimensions of self-reported mental health among individuals in higher education and individuals *not* in higher education, separated by gender. Because the mental health variables were assessed on different scales, we rescaled them so that they had the same lower and upper limits, making them directly comparable on a scale now ranging from 0 to 5 for all variables. The horizontal line within the boxes represents the median; the box edges represent the 1st and 3rd quartiles; the end of the whiskers equal (Q3 + 1.5 * IQR) and (Q1 − 1.5 * IQR). Observations outside the whiskers are shown as dots

To get a better understanding of the mental health among both groups and fully address our first research question, we also considered gender differences and visualized the distributions of each dimension of mental health across groups. The box plots in Fig. 1 illustrate these distributions. *Among individuals in higher education*, we found significant gender differences in mental health, *F* (5, 2181) = 30.31, *p* < 0.001. Post hoc analyses indicated that self-esteem was slightly but significantly lower among women than among men (*M* = 3.95, *SD* = 0.70 versus *M* = 4.08, *SD* = 0.64, *p* < 0.001), that self-efficacy was significantly lower among women than among men (*M* = 3.38, *SD* = 0.68 versus *M* = 3.64, *SD* = 0.66, *p* < 0.001), and that levels of negative affectivity were significantly higher among women than among men (*M* = 1.73, *SD* = 0.87 versus *M* = 1.44, *SD* = 0.81, *p* < 0.001). *Among individuals who never enrolled in higher education*, there were also significant gender differences in mental health*, F* (5, 4110) = 31.56, *p* < 0.001. Post hoc analyses revealed that self-esteem was significantly higher among men than among women (*M* = 4.02, *SD* = 0.65 versus *M* = 3.90, *SD* = 0.68, *p* < 0.001). Self-efficacy was significantly higher among men than among women (*M* = 3.65, *SD* = 0.68 versus *M* = 3.44, *SD* = 0.67, *p* < 0.001). Affectivity was more negative among women than among men (*M* = 1.63, *SD* = 0.90 versus *M* = 1.43, *SD* = 0.84, *p* < 0.001). In sum, although gender differences in mental health were relatively small among both students and nonstudents, some gender differences were noteworthy nonetheless, including the higher levels of self-efficacy among men than women in both groups and the more negative affectivity among women than among men in both groups.

### Predicting degree attainment as a function of distinct dimensions of mental health

Before we turn to the second research question about the extent to which mental health predicts higher education degree attainment, note the bivariate correlations between distinct dimensions of mental health and students’ degree attainment (Table 3). Higher education degree attainment was significantly positively correlated with positive attitude towards life (*r* = 0.10, *p* < 0.001) and with self-esteem (*r* = 0.09, *p* < 0.001). Degree attainment was neither significantly correlated with self-efficacy (*r* = 0.02, *p* > 0.05) nor with perceived stress (*r* = 0.00, *p* > 0.05). Finally, degree attainment was weakly negatively correlated with negative affectivity (*r* = −0.06, *p* < 0.05).

We estimated two models to ascertain the role of mental health in degree attainment, thereby addressing *Research Question 2* about the predictive power of distinct dimensions of mental health for degree attainment among higher education students (Models 1 and 2 in Table 4). In Model 1, we estimated higher education students’ probability of attaining a degree solely as a function of the covariates, whereas in Model 2 we added all mental health variables. This allowed us to evaluate whether adding the mental health variables improved the fit of the model, beyond simply estimating the likelihood of degree attainment as a function of mental health variables. A chi-square difference test indicated that Model 2 exhibited a significantly better fit with the empirical data relative to Model 1, ∆χ^2^ (5) = 32.36, *p* < 0.001, indicating that the mental health dimensions examined here contributed to explaining the variance in individuals’ probability of attaining a degree.

**Table 4:** Coefficients from Linear Models Estimating the Probability of Degree Attainment

	Model 1	Model 2	Model 3
	*β* (SE)	*β* (SE)	*β* (SE)
Male	−0.04 (0.02)*	−0.04 (0.02)	−0.16 (0.26)
Immigrant	−0.01 (0.04)	−0.00 (0.03)	−0.01 (0.03)
Age (in months)	−0.00 (0.00)	−0.00 (0.00)	−0.00 (0.00)
Socioeconomic status	0.00 (0.00)	0.00 (0.00)	0.00 (0.00)
Reading performance at age 15	0.00 (0.00)	0.00 (0.00)	0.00 (0.00)
Math performance at age 15	0.00 (0.00)	0.00 (0.00)	0.00 (0.00)
Science performance at age 15	0.00 (0.00)	0.00 (0.00)	0.00 (0.00)
Academic self-concept at age 15	0.02 (0.01)	0.02 (0.01)	0.02 (0.01)
Study effort at age 15	−0.00 (0.01)	−0.01 (0.01)	−0.00 (0.01)
Control expectation at age 15	0.00 (0.01)	0.00 (0.01)	0.00 (0.01)
University	−0.01 (0.02)	−0.00 (0.02)	−0.00 (0.02)
*Dimensions of mental health*			
Positive attitude towards life	–	0.06 (0.02)**	0.06 (0.03)*
Self-esteem	–	0.03 (0.03)	0.00 (0.03)
Self-efficacy	–	−0.06 (0.03)*	−0.03 (0.04)
Negative affectivity	–	−0.01 (0.02)	−0.03 (0.02)
Perceived stress	–	0.02 (0.01)	0.02 (0.02)
*Interactions*			
Male * Positive attitude towards life	–	–	0.00 (0.04)
Male * Self-esteem	–	–	0.06 (0.05)
Male * Self-efficacy	–	–	−0.06 (0.06)
Male * Negative affectivity	–	–	0.04 (0.04)
Male * Perceived stress	–	–	−0.01 (0.03)
*Indices of model fit*			
Log-likelihood	−912.10	−895.92	−892.72
AIC	1850.20	1827.84	1831.44
BIC	1923.46	1929.28	1961.05

*β* linear probability coefficients, *SE* robust standard errors

***p < 0.001, **p < 0.01, *p < 0.05

Model 2 suggests that individuals with a more positive attitude towards life were more likely to obtain a degree than their counterparts with less positive attitudes towards life, net of all other variables (*p* < 0.01). Moreover, individuals with higher levels of self-efficacy were less likely to obtain a degree than their counterparts with lower levels of self-efficacy (*p* < 0.05), but note that this negative association only emerged in the regression model (the zero-order correlation between the two variables was insignificant). Finally, Model 2 (Table 4) shows that self-esteem, negative affectivity and perceived stress were not significantly related to individuals’ probability of attaining a degree, when controlling for all other variables.

### The role of gender

**Fig. 2:**
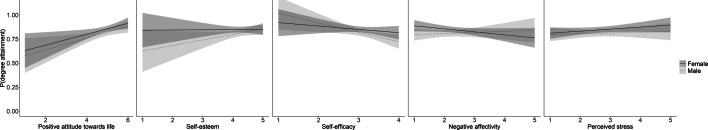
Predicted probabilities of attaining a degree in higher education as a function of distinct dimensions of mental health and gender, with 95% confidence intervals

To address *Research Question 3* on whether gender moderates the links between mental health and degree attainment, we added interaction terms to the model (see Model 3 in Table 4). The results generated by running this model indicated that gender did not significantly moderate any of the links between the various dimensions of mental health and degree attainment (with all *p* > 0.05). Importantly, the interactions proved insignificant even when we added them one by one to the model. A likelihood ratio (chi-square) test assessing differences in model fits also suggested that Model 3 (with interaction terms) did not fit the empirical data better than Model 2 (without interaction terms), ∆χ^2^ (5) = −6.4, *p* > 0.05. Thus, although we found some evidence of gender differences in the average levels of self-esteem, self-efficacy, negative affectivity, and perceived stress (see Fig. 1), we did not find evidence of significant gender differences in the associations between mental health and higher education degree attainment. Figure 2 plots the nonsignificant interactions (solid lines for female respondents, dashed lines for male respondents). More specifically, Fig. 2 illustrates how the predicted probabilities of attaining a degree varied as a function of the five dimensions of mental health examined here. We plotted those predicted probabilities separately for men and women. The 95% confidence intervals indicate the lower and upper bounds within which the true population scores were likely to lie. To discuss an example, let us focus on the left-most panel, which shows individuals’ probability of being awarded a degree as a function of their attitude towards life. On average, female respondents with the lowest reported value on the scale measuring attitude towards life exhibited a roughly 63% predicted probability of being awarded a higher education degree, but this probability increased as the attitude towards life became more positive. Among female respondents with the most positive attitudes towards life, the probability of attaining a degree was estimated to be around 90%. All panels illustrate that gender did not significantly moderate the links between mental health and degree attainment.

## Discussion

Given concerns about mental ill-health among students in higher education (e.g., Macalli et al. 2021), we compared the mental health status of higher education students to that of individuals who did not pursue higher education. Moreover, we examined whether diverse dimensions of mental health were implicated in processes underlying degree attainment among higher education students and whether gender moderated the longitudinal associations between mental health and higher education degree attainment.

### Mental health of students and nonstudents

Findings suggested that the mental health status of higher education students was quite similar to that of their nonstudent counterparts. Both groups reported a similarly positive attitude towards life. Among students, self-esteem was slightly higher, self-efficacy was slightly lower, and their affectivity was slightly more negative than that of nonstudents. The most important difference between students and nonstudents pertained to the level of perceived stress, which was considerably higher among students. Students in higher education may face academic pressure and unique challenges related to the academic environments they are in (Pedrelli et al. 2015). Many students in higher education take education quite seriously and show high levels of study engagement (Salmela-Aro and Read 2017). The demands that their programs impose on them may be an important source of stress (see also Saleh et al. 2017). However, it is important to remember that only a subgroup of nonstudents reported their perceived stress levels. Thus, considering our findings on the whole, we note that students and their nonstudent counterparts had relatively similar mental health needs.

### The longitudinal links between mental health and degree attainment

We found evidence that the distinct dimensions of mental health were differentially related to subsequent degree attainment. As such, our results are in line with prior evidence indicating that diverse aspects of psychological functioning have a disparate predictive power for educational attainment (Burger et al. 2020; Veldman et al. 2014). More specifically, our study confirms previous research by suggesting that a positive attitude towards life has a positive impact on attainment processes (De Neve and Oswald 2012). In contrast, we found a negative link between self-efficacy and subsequent attainment, net of all the other effects. There are various fields of action in which individuals can capitalize on self-efficacy. The capacity to persevere in goal-oriented activities appears to be critically important for young adults at a time when socioeconomic trajectories in both education and work are being formed (cf., Mortimer et al. 2016). Yet, both education- and work-related goal-setting may depend on efficacy beliefs. Self-efficacy in this early phase of career establishment might, in fact, also be an important psychological resource in the transition to job training or a successful work career. Prior research has shown that self-efficacy and related attributes such as mastery beliefs foster the acquisition of adult roles, including success in entering the labor market (Reynolds et al. 2007; Vuolo et al. 2012). Importantly, however, general perceived self-efficacy may exert differential effects in diverse domains (Grabowski et al. 2001). Hence, domain-specific academic self-efficacy might be more predictive of subsequent educational attainment than global self-efficacy (Honicke and Broadbent 2016), which was the focus of the present study. Finally, it is important to remember that there was no significant bivariate correlation between self-efficacy and degree attainment. The insignificant zero-order correlation points to the possibility of overcontrol or collider bias in the linear probability models and suggests that the negative effect of self-efficacy on educational attainment identified here might reasonably be interpreted as practically negligible.^3^

Further findings suggest that self-esteem was not significantly related to individuals’ probabilities of attaining a degree. This result is at odds with studies that found positive effects of self-esteem on educational outcomes (Booth and Gerard 2011; Marsh and O’Mara 2008) and it aligns, in contrast, with studies that found only weak or no significant effects of self-esteem on later life course outcomes (Baumeister et al. 2003; Boden et al. 2008). Overall, these mixed findings might also be explained partially by the fact that self-esteem can be both a cause and a consequence of educational outcomes (see also Burger et al. in press).

Moreover, our models indicate that negative affectivity was not significantly related to the likelihood of subsequently attaining a degree, although researchers have claimed that negative affectivity puts individuals at risk of functioning poorly in psychological and social domains (Norton and Paulus 2016; Watson and Naragon-Gainey 2014). It is important to stress, however, that we did not investigate negative affectivity in a clinical sample. It is conceivable that a stable tendency to experience aversive emotional states in clinical forms may have more severe repercussions for attainment processes. It is also possible that individuals who exhibit high levels of negative affectivity across time and regardless of the situation are at higher risk of failing to attain a degree. Here, study participants reported their affectivity for a one-month period in each of three waves (2005, 2006, and 2007). It would be important for future research to focus more specifically on individuals who consistently report highly negative affect over long periods of time.

Finally, our findings indicated that perceived stress was not significantly related to students’ degree attainment. We propose two explanations for this finding. First, students may already have learned to cope with stressful events (Skinner and Zimmer-Gembeck 2007); hence, they might be capable of handling stress effectively when necessary. Second, some students may suffer due to perceived stress, whereas others may benefit from it. Accordingly, perceived stress might impair educational performance among some students but improve it among others. In fact, perceived stress might stimulate learning activities and study motivation, spurring some students on to better educational performance (Bower et al. 2008) and hence counteracting potentially negative impacts of stressful experiences (Folkman 2008; Park et al. 1996).

Note also that, among higher education students, the probability of attaining a degree was not significantly related to sociodemographic variables or other covariates that were measured in adolescence, at age 15. Considering the nonsignificant associations between these key control variables and degree attainment, the significant longitudinal link between students’ attitudes towards life and degree attainment is noteworthy. It indicates that a positive attitude may benefit academic educational trajectories and, by extension, shape later life-course outcomes.

### The role of gender in the links between mental health and degree attainment

We did not find evidence that gender moderates any potential influence of mental health on degree attainment. This corroborates conclusions from prior research that mental health is equally important for the educational outcomes of both genders (Brännlund et al. 2017; Deighton et al. 2018; Ding et al. 2009; Esch et al. 2014; Hjorth et al. 2016), further calling into question theories that posit gender-specific skills and strategies to deal with poor mental health (Eschenbeck et al. 2007; Hampel and Petermann 2006). Although our study cannot determine whether young men and women respond in different ways to specific developmental tasks and psychological distress (cf. Dedovic et al. 2009), the evidence presented here suggests that, as far as educational attainment is concerned, if anything, poor mental health is likely to harm young men and women in equal measure, and it is therefore important to attend to the specific needs of students who exhibit poor mental health regardless of gender.

### Limitations

This study is not without limitations. First, we acknowledge that it is difficult to disentangle cause and effect or to exclude reciprocal associations between dimensions of mental health and educational outcomes (Bortes et al. 2021; Marsh and O’Mara 2008; Steinmayr et al. 2016). Better mental health may help students to perform well. At the same time, success in education may create feedback loops that boost the effects of mental health on educational performance. In the current study, we used a longitudinal design to assess whether indicators of mental health served as precursors to higher education degree attainment, after we had controlled for observable potential confounders. It would not have been possible to induce a given mental health state over multiple years to evaluate its effects on higher education degree attainment experimentally. Accordingly, our longitudinal observational data and current findings are of substantial value. Second, we focused on variations in mental health in the general population. In clinical samples, the impacts of poor mental health on various life-course outcomes, including educational attainment, would likely be more substantial. Third, the present findings are based on a nationally representative sample and, as such, are representative of a specific cohort of individuals in Switzerland. However, they cannot be generalized to other cohorts or countries, where there may be greater differences in the mental health profiles of students and nonstudents and where the higher education system may not resemble the Swiss one. Finally, more research will be needed to assess perceived stress and its consequences in various domains of young adults’ lives.

## Conclusion

We found that, overall, the mental health of higher education students was relatively similar to that of their nonstudent counterparts. However, students exhibited slightly higher self-esteem, slightly weaker self-efficacy, more negative affectivity, and higher levels of perceived stress. The effects of different facets of mental health on higher education degree attainment were mostly statistically and/or practically insignificant. A noteworthy exception was individuals’ attitudes towards life. Students with a more positive attitude towards life were substantially more likely to subsequently attain a degree from higher education than their counterparts with a less positive attitude, even when controlling for observable potential confounders. Young men and women had somewhat different mental health needs; however, gender did not significantly moderate any links between the distinct dimensions of mental health and individuals’ subsequent degree attainment. From the evidence presented here, it thus seems implausible to assume considerable gender differences in the effects of mental health problems on educational careers. Hence, we conclude that it is critical to attend to the mental health needs of both male and female students alike to enable them to achieve their full potential in higher education. We also recommend, however, that future research examine mental health problems of different severity, including clinically relevant symptoms of mental disorders, and how those might disrupt educational careers.

### Supplementary Information

Appendix A

## Footnotes

1 Note that we did not consider mental disorders—clinically relevant disturbances in behavior, thinking, emotional regulation, or relationships with other people (such as anxiety, substance use, psychotic disorders, major depression, eating disorders, bipolar disorder, personality disorders, or other disorders).

2 PISA used a two-stage stratified sampling design. In the first stage, schools were sampled with probabilities proportional to the size of the student body. In the second stage, students were sampled within schools.

3 This interpretation is also plausible because the effect of self-efficacy becomes insignificant when we additionally control for the school tracks that students attended at the lower-secondary level (academically demanding versus less demanding tracks), whereas the effects of the other mental health dimensions remain unchanged.
