# Supplementary material for: Mental health, gender, and higher education attainment
Source: Z Erziehwiss. 2023 Sep 6;27(1):89–122. doi: 10.1007/s11618-023-01187-3 (PMC10942912; doi:10.1007/s11618-023-01187-3)
Supplement: Supplementary file 1 — Appendix A [file 11618_2023_1187_MOESM1_ESM.docx]

| **Appendix A**  **Table A1** Odds Ratios from Logistic Regressions Estimating the Likelihood of Degree Attainment | | | | | | |
| --- | --- | --- | --- | --- | --- | --- |
|  | Model 1 | | Model 2 | | Model 3 | |
|  | Odds ratio | 95% CI | Odds ratio | 95% CI | Odds ratio | 95% CI |
| Male | 0.75* | [0.57; 0.99] | 0.76 | [0.57; 1.01] | 0.50 | [0.97; 1.00] |
| Immigrant | 0.93 | [0.59; 1.46] | 1.02 | [0.64; 1.61] | 1.01 | [0.85; 1.22] |
| Age (in months) | 0.99 | [0.97; 1.00] | 0.99 | [0.97; 1.00] | 0.98 | [0.64; 1.60] |
| Socioeconomic status (HISEI) | 1.01 | [1.00; 1.02] | 1.01 | [1.00; 1.02] | 1.01 | [0.02; 14.64] |
| Reading performance at age 15 | 1.00 | [1.00; 1.00] | 1.00 | [1.00; 1.00] | 1.00 | [1.00; 1.02] |
| Math performance at age 15 | 1.00 | [1.00; 1.00] | 1.00 | [1.00; 1.00] | 1.00 | [1.00; 1.00] |
| Science performance at age 15 | 1.00 | [1.00; 1.00] | 1.00 | [1.00; 1.00] | 1.00 | [1.00; 1.00] |
| Academic self-concept at age 15 | 1.18* | [1.00; 1.39] | 1.14 | [0.96; 1.35] | 1.14 | [1.00; 1.00] |
| Study effort at age 15 | 0.98 | [0.85; 1.14] | 0.97 | [0.84; 1.12] | 0.97 | [0.97; 1.35] |
| Control expectation at age 15 | 1.00 | [0.84; 1.20] | 1.02 | [0.85; 1.22] | 1.02 | [0.84; 1.12] |
| University | 0.96 | [0.74; 1.23] | 0.97 | [0.75; 1.26] | 0.97 | [0.75; 1.26] |
| *Dimensions of mental health* |  |  |  |  |  |  |
| Positive attitude towards life |  |  | 1.47** | [1.15; 1.89] | 1.50* | [1.04; 2.16] |
| Self-esteem |  |  | 1.19 | [0.85; 1.66] | 1.01 | [0.65; 1.57] |
| Self-efficacy |  |  | 0.67 | [0.44; 1.01] | 0.80 | [0.46; 1.38] |
| Negative affectivity |  |  | 0.91 | [0.71; 1.15] | 0.78 | [0.57; 1.08] |
| Perceived stress |  |  | 1.12 | [0.93; 1.35] | 1.19 | [0.91; 1.56] |
| *Interactions* |  |  |  |  |  |  |
| Male*Positive attitude towards life |  |  |  |  | 0.98 | [0.60; 1.61] |
| Male*Self-esteem |  |  |  |  | 1.39 | [0.73; 2.64] |
| Male*Self-efficacy |  |  |  |  | 0.67 | [0.30; 1.51] |
| Male*Negative affectivity |  |  |  |  | 1.38 | [0.86; 2.22] |
| Male*Perceived stress |  |  |  |  | 0.88 | [0.57; 1.34] |
| *Indices of model fit* |  |  |  |  |  |  |
| McFadden’s R-squared | 0.02 |  | 0.03 |  | 0.04 |  |
| Nagelkerke’s R-squared | 0.02 |  | 0.04 |  | 0.04 |  |
| Cox and Snell R-squared | 0.03 |  | 0.06 |  | 0.06 |  |
| Log-likelihood | -938.13 |  | -922.88 |  | -919.51 |  |
| *Note*. CI = confidence intervals. The three R-squared measures mentioned here are pseudo R-squareds and cannot be interpreted like R-squareds from an OLS regression. This is because the model coefficients in logistic regression are not calculated to minimize variance but are instead maximum likelihood estimates produced through an iterative process. When comparing two models based on the same data, the model with the higher pseudo R-squared value represents the model with the greater likelihood. While the pseudo R-squareds do not allow for unambiguously establishing whether Model 2 or Model 3 is more likely, they show that Models 2 and 3 are both more likely than Model 1. *** *p* < .001, ** *p* < .01, * *p* < .05. | | | | | | |
